# The complete mitochondrial genome sequence of *Schizothorax dulongensis* (Cypriniformes, Cyprinidae) from Irrawaddy River in China

**DOI:** 10.1080/23802359.2021.1959454

**Published:** 2021-08-18

**Authors:** Yuchao Song, Songtao Li, Haoran Gu, Zhijian Wang

**Affiliations:** Key Laboratory of Freshwater Fish Reproduction and Development (Ministry of Education), Key Laboratory of Aquatic Science of Chongqing, School of Life Sciences, Southwest University, Chongqing, China

**Keywords:** *Schizothorax dulongensis*, mitochondrial genome, phylogeny

## Abstract

*Schizothorax dulongensis* Huang (1985) is an endemic species in China, which is mainly distributed in the Irrawaddy River and its tributaries. In this study, the complete mitochondrial genome was sequenced. Its length is 16,579 bp, containing 22 tRNAs, two rRNAs, and 13 PCGs. The phylogenetic results showed that *S. dulongensis* and these species from Brahmaputra River clustered a clade, and supported the Brahmaputra River and the Irrawaddy River have been reorganized of drainage.

## Introduction

*Schizothorax* fishes are mainly distributed on the Qinghai-Tibet Plateau and surrounding water systems, and they are important organisms for studying the geological evolution of the Qinghai-Tibet Plateau. Most of the complete mitochondrial genome sequences of *Schizothorax* fishes from other water systems have been reported, but these species from the Irrawaddy River have not been reported, which limits their biogeographical research. Therefore, in this study, the whole mitochondrial genome of *S. dulongensis* from Irrawaddy River was sequenced and analyzed.

*Schizothorax dulongensis* belongs to the Cypriniformes, Cyprinidae, Schizothoracinae, and *Schizothorax* Heckel. The samples were collected from the upper Irrawaddy River (Yunnan, China; 27.843°N, 98.327°E), and a specimen was deposited at Southwest University Museum of Zoology under the voucher number DL-SCH-1-4-20200918 (the contact person is the corresponding author). This mitogenome was submitted to GenBank database with accession No. MW748176.

## Material and method

The DNA extraction kit (DC102 FastPure CellTissue DNA Isolation Mini Kit) was used to extract the genomic DNA from *S. dulongensis*’s pelvic fin. The paired-end (2 × 150 bp) library was constructed and then sequenced on the Illumina Hiseq 5000 platform. The mitochondrial genome was assembled by NOVOPlasty (https://github.com/ndierckx/NOVOPlasty) with *Schizothorax davidi* (GenBank: KM879227) (Wang et al. 2016) as the initial reference, and online tool MITOS (http://mitos2.bioinf.uni-leipzig.de/index.py) was used to annotate the mitochondrial genome. The ML phylogenetic tree using 13 protein-coding gene sequences was constructed by IQ-TREE with default setting and model (Trifinopoulos et al. 2016).

## Results

The complete mitochondrial genome is 16,579 bp in length, including 22 transfer RNAs (tRNAs) with a total length of 1562 bp, two ribosomal RNAs (rRNAs) with a total length of 2592 bp, and 13 protein-coding genes (PCGs) with a total length of 11,415 bp. The *ND6* and eight tRNAs are encoded on the light (L) strain, and the other genes are encoded on the heavy (H) strain. Among PCGs, the start codon of *COX1* is GTG, the start codon of other PCGs are ATG; the stop codon of *ND2*, *ATP8* and *ND3* are TAG and the rest mostly are TAA as the stop codon. The incomplete stop codon T–– is found in *COX2*, *ND4* and *CYTB*. The nucleotide composition of the whole mitochondrial genome of *S. dulongensis* includes 30.21% A, 25.64% T, 17.38% G, and 26.77% C.

**Figure 1.**
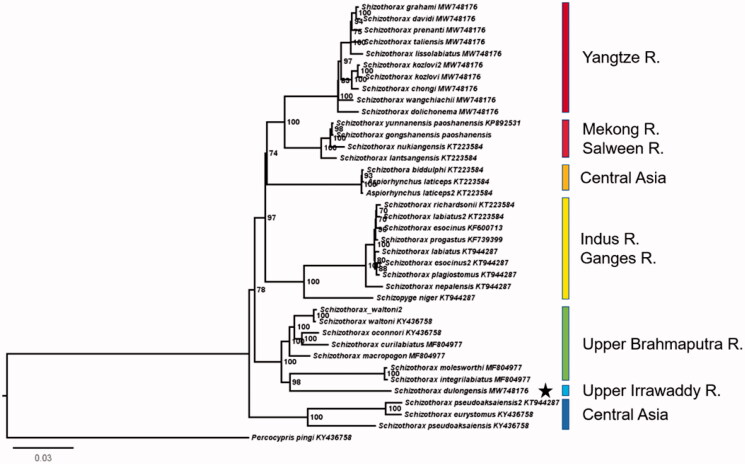
Phylogenetic relationship showing the relationship of *Schizothorax* fishes. Numbers in front of each node indicates the bootstrap support value.

The phylogenetic results showed that *S. dulongensis* and these species from Brahmaputra River clustered a clade, and have clearly differentiated from species in other water systems. This showed the evidence that the Brahmaputra River and the Irrawaddy River had been reorganized of drainage (Figure 1), and the results supported the conclusions of previous geological studies (Clark et al. 2004).

## Data Availability

The genome sequence data that support the findings of this study are openly available in GenBank of NCBI at (https://www.ncbi.nlm.nih.gov/) under the accession no. MW748176.

## References

[CIT0001] Clark MK, Schoenbohm LM, Royden LH, Whipple KX, Burchfiel BC, Zhang X, Tang W, Wang E, Chen L. 2004. Surface uplift, tectonics, and erosion of eastern Tibet from large-scale drainage patterns. Tectonics. 23(1).

[CIT0002] Trifinopoulos J, Nguyen LT, von Haeseler A, Minh BQ. 2016. W-IQ-TREE: a fast online phylogenetic tool for maximum likelihood analysis. Nucleic Acids Res. 44(W1):W232–W235. 27084950 10.1093/nar/gkw256 PMC4987875

[CIT0003] Wang T, Jiao WL, Zhang YP, Lou ZY, Du YY, Yang ZY. 2016. Characterization of the complete mitochondrial genome of *Gymnocypris chilianensis* (Teleostei: Cypriniformes: Cyprinidae). Mitochondrial DNA A DNA Mapp Seq Anal. 27(6):4015–4017. 25629501 10.3109/19401736.2014.1003822

